# Validation and translation of the Hungarian version of the Australian Pelvic Floor Questionnaire (APFQ-H)

**DOI:** 10.1007/s00192-022-05322-2

**Published:** 2022-08-16

**Authors:** Márta Hock, István Tiringer, Eszter Ambrus, Zoltán Németh, Bálint Farkas

**Affiliations:** 1 Institute of Physiotherapy and Sport Sciences, Faculty of Health Sciences, University of Pécs, 3 Vörösmarty str., Pécs, 7621 Hungary; 2 Institute, of Behavioural Sciences, Medical School, University of Pécs, 12 Szigeti str., Pécs, 7623 Hungary; 3 Harkány Thermal Rehabilitation Center, 1 Zsigmondy str., Harkány, 7815 Hungary; 4 Department of Gynecology, Hospital St. John of God, 1 Johannes von Gott-Platz, 1020 Vienna, Austria; 5 Department of Obstetrics and Gynecology, University of Pécs, Pécs, Hungary; 6 Member of the MTA-PTE Human Reproduction Scientific Research Group, Hungarian Academy of Sciences, 17 Édesanyák str., Pécs, Budapest 7624 Hungary

**Keywords:** Validation study, Validity, Patient health questionnaire, Pelvic floor disorders, Quality of life

## Abstract

**Introduction and hypothesis:**

The aims of the study were the translation, cultural adaptation, and validation of self-administered Australian Pelvic Floor Questionnaire (APFQ) on a Hungarian population.

**Methods:**

The validation was performed in 294 women over 18 who agreed to answer the Hungarian version of the APFQ. The validation of the questionnaire included evaluation of content/face validity, internal consistency, construct validity, test-retest reproducibility, discriminant validity and convergent validity.

**Results:**

Acceptable and good internal consistency was observed in all four dimensions [McDonald’s ω (95% confidence interval) coefficients were > 0.7 for each dimension: bladder 0.888, bowel 0.790, prolapse 0.895 and sexual function 0.738]. Test-retest analyses revealed high reproducibility with intraclass correlation coefficients (bladder 0.83, bowel 0.92, prolapse 0.96 and sexual function 0.87). Prolapse symptom score correlated significantly with Pelvic Organ Prolapse Quantification (POP-Q), and bladder score correlated significantly with the results of the International Consultation on Incontinence Questionnaire-Urinary Incontinence Short Form (ICIQ UI SF) (convergent validity). Scores distinguished between patients with pelvic floor disorders and controls (high discriminant validity).

**Conclusions:**

Hungarian version of the self-administered APFQ is a reliable and valid instrument for evaluating symptom severity and impact of pelvic floor dysfunction on the quality of life of Hungarian women.

**Supplementary Information:**

The online version contains supplementary material available at 10.1007/s00192-022-05322-2.

## Introduction

Pelvic floor disorders (PFD) in women such as urinary incontinence (UI), faecal incontinence (FI), pelvic organ prolapse (POP), sexual dysfunction and other urogenital symptoms are common [1]. It is estimated that women have a one in four lifetime risk of experiencing a PFD [2]. Annual ambulatory costs of PFDs are estimated upwards of $300 million, and > $12 billion is spent each year on the surgical and nonsurgical management of stress urinary incontinence (SUI) [3, 4]. It is well known that women with urinary incontinence (UI) are more likely to have concomitant anal incontinence (AI) than those without UI [5]. The prevalence of double incontinence was 9.3%, respectively, among those aged ≥ 65 years and over [6]. Prevalence of triple PFDs is 24% and in case of four types is 8% among postnatal women according to Durnes et al. [7]. These disorders may severely influence women’s quality of life (QOL) [8]. Many women suffer from PFDs, incorrectly believing that these are a normal consequence of ageing. Health care providers can screen women for PFDs and help them find appropriately trained physicians, but multidisciplinary team-approached care for a woman with pelvic floor conditions may be more useful yet [9]. The situation of health care in Hungary is even worse, there are no multidisciplinary teams with adequate questionnaires for the proper care of patients, and we do not know exactly how many patients suffer from multiple incontinence or what types of prolapse they are struggling with. When solving the problem, an appropriate questionnaire may be the first step. The Australian Pelvic Floor Questionnaire (APFQ) [10] comprehensively assesses four domains, bladder, bowel, prolapse and sexual symptoms, their severity, their bother and impact on quality of life. A further benefit is that it is a self-administered questionnaire [11]. The APFQ was initially developed in English and adapted into German, French, Turkish, Arabic, Serbian and Chinese [12–17]. The aim of this study was to translate the self-administered version of the Australian Pelvic Floor Questionnaire into Hungarian and to evaluate its psychometric properties in Hungarian women with pelvic floor disorders.

## Materials and methods

### Participants

A total of 294 participants were enrolled. Volunteers, women aged between 18 and 85 years, were recruited in the current study. Patients with pelvic floor disorders (*n* = 109) were included in this study if they were diagnosed with POP alone or together with UI at the Clinical Centre of the University of Pécs, Department of Obstetrics and Gynecology, Department of Gynaecology, Thermal Rehabilitation Centre Harkány and the Ladypower® Private Clinic (Győr) between January 2019 and December 2020 in Hungary. All patients demonstrated symptomatic (bulge sensation in their vagina with or without symptoms of urinary, bowel or sexual dysfunction) stage ≥ 1 POP of the anterior, middle, and/or posterior compartments of the vagina. All of the participating patients were examined according to the guidelines established by the International Urogynecological Association. The patients enrolled in the survey were informed that their participation was anonymous and completely voluntary. All patients provided their written informed consent to participation. After a short personal interview focusing on the general health conditions and medical history (previous surgeries, pharmaceutical or hormone treatments, co-morbidity, and data related to childbirth were registered), the specialists determined the POP quantification during gynaecological examination. After that the participants filled out the questionnaires. The exclusion criteria were dementia, prior or current malignancy, neurological (dystrophy, myositis, myopathy and neuropathy) and psychiatric diseases (depression, schizophrenia, mental disabilities), addiction to psychoactive substances and/or alcohol, pregnancy or being within 12 months postpartum, lack of an informed written consent and illiteracy.

We also recruited healthy women, who attended regular annual gynaecological check-ups to serve as a control group (*n* = 185) on different online interfaces under the same demographic conditions. The data were collected through online surveys, designed using Google survey. Aiming to reach a bigger study population, we sent the survey to employees at Thermal Rehabilitation Centre Harkány, their friends and relatives through emails and on Facebook.

This study was approved by the Institutional Review Board of the Faculty of Medicine, University of Pécs, in 2019 (no.7950) in accordance with the 2008 Helsinki Declaration.

The validation of the Hungarian version of the Australian pelvic floor questionnaire included evaluation of content/face validity, internal consistency, factorial validity, test-retest reproducibility, discriminant validity and convergent validity.

### Validation

Two questionnaires [International Consultation on Incontinence Questionnaire-Urinary Incontinence Short Form (ICIQ), Pelvic Organ Prolapse/Urinary Incontinence Sexual Questionnaire (PICQ–IR)] and Pelvic Organ Prolapse Quantification (POP-Q) were used for data collection. Data on socio-demographic characteristics (highest level of education, occupation, place of residence), anthropometric (weight, height) and obstetrical and gynaecological anamnesis were collected as well.

### Discriminant validity

To examine whether the Hungarian version of the APFQ can detect differences between a general/healthy population and a clinical population, we used the data of a group of women with pelvic organ prolapse (POP), which is known to be correlated with decreased sexual quality of life because of the altered genital anatomy and involuntary coital urine incontinence [18]. All women had ≥ stage 1 POP of the anterior, middle or posterior compartment, or a combination of them. All reported a sensation of a bulge in the vagina with or without symptoms of urinary, bowel or sexual dysfunction. (All methods, definitions and units conform to the standards set by the International Urogynaecology Association and the International Continence Society [19].)

### Original Australian Pelvic Floor Questionnaire (APFQ)

The self-administered APFQ consists of four domains, namely bladder function (15 items), bowel function (12 items), prolapse symptoms (5 items) and sexual function (10 items) [11]. Thirty-eight of the items assess pelvic floor symptoms and four questions assess the bother caused by the symptoms in all areas. The last question in each domain of the questionnaire was the evaluation of bother, “How much do your bladder/bowel/prolapse/ sexual symptoms bother you?” If the participant chose any of “a little”, “quite a lot” and “very much”, then it was regarded as bother and the variable dichotomised accordingly [20]. Most of the items in the questionnaire use a 4-point scoring system apart from defecation frequency, bowel consistency, sufficient lubrication and reason for sexual abstinence. The symptom scores and bothersome scores were added to a global score. The score of relevant questions is divided by the total score for each domain and multiplied by 10, with a value of 0–10 for each domain and a global maximum pelvic floor dysfunction score of 40. The higher the score is, the more severe the pelvic floor symptoms. If women are not sexually active because of pelvic floor dysfunction, they will get a score of 8.5 (18/21) in the sexual domain. If a woman is not sexually active, the maximum score is 30.

Since there is no validated Hungarian questionnaire that measures the same context as the APFQ available, the validated ICIQ SF, PISQ-IR questionnaires and POP quantification were applied as a benchmark.

### International Consultation on Incontinence Questionnaire-Urinary Incontinence Short Form (ICIQ-UI SF)

The symptom questionnaire (ICIQ-UI SF) consists of three scored questions concerning frequency, amount of leakage and overall inconvenience. In addition, there is a fourth unscored diagnostic question aimed to determine the type of urinary incontinence [21]. In the current study we used the validated Hungarian version of ICIQ-UI SF [22].

### Pelvic Organ Prolapse/Urinary Incontinence Sexual Questionnaire IUGA-revised (PISQ-IR)

The PISQ-IR is a self-administered validated questionnaire that contains 20 items and aims to assess the sexual functions in women with POP. In the current study, we used the validated Hungarian version of PISQ-IR [23–25].

### Pelvic Organ Prolapse Quantification (POP-Q)

Pelvic Organ Prolapse Quantification system (POP–Q), designed by the The International Continence Society (ICS), the American Urogynecologic Society (AUGS) and the Society of Gynecologic Surgeons in 1996, refers to an objective, site-specific system for describing, quantifying and staging pelvic support in women, which was used in our current study to assess POP severity [26].

The validated self-administered anal/faecal incontinence questionnaire is not available in Hungary.

### Translation of the APFQ into Hungarian (APFQ-H)

After obtaining the agreement from the author (Kaven Baessler) to perform the cultural adaptation of the APFQ into Hungarian, the linguistic validation was carried out in accordance with the guidelines of linguistic validation processes. The original APFQ was translated from English into Hungarian by two physiotherapists, who are fluent in both English and Hungarian (version 1). The back translation of the APFQ-H into the original language was carried out by an independent bilingual physician. Finally, it was also reviewed by the study researchers to obtain a reliable translation. Then, a face-to-face interview was also conducted with five women to check for any difficulties in understanding and interpreting the questions. No major difficulties were noted (version 2) [27].

### Content/face validity

To test the content validity, expert consultation was performed with three obstetrics and gynaecologists and two specialist physicians to evaluate the translated questionnaire. After that ten volunteer participants in our test group were asked to fill out the face-to-face pretest of the questionnaire, and during the consultation this was checked with them to assess the clarity of the items. There were not significant deficits or notes, so the research group accepted the final Hungarian version of the self-administered APFQ.

### Factorial validity

Factorial validity was controlled by the application of confirmatory factor analysis (CFA). Confirmatory factor analysis is a structural equation modelling technique used to determine the fit between an assumed factor structure and empirical data. For each scale of the APFQ, a separate measurement model was developed to examine the dimensionality of the scales. In the case of the Bowel and Sex subscales where fit indices were less than optimal, exploratory factor analysis was performed to investigate the structure of the answers.

### Convergent and divergent validity

We investigated how APFQ is related to same domains/status of the ICIQ and PISIQ-IR questionnaires and POPQ stages.

### Discriminant validity

To evaluate discriminant validity, the questionnaire was completed by 109 patients with at least one symptom related to pelvic floor dysfunction and 185 women who did not report PFD symptoms.

### Reliability

#### Test-retest reliability

The final approved version was tested in a pilot study on 31 women prior to the study. During the first visit, we conducted a face-to-face interview and collected demographic data. These participants completed the same questionnaire twice. Duration of the test-retest period was 2 weeks for all patients. (No adaptive treatment was offered to any of these patients during this interval.) Finally, the test-retest correlations were evaluated by applying intraclass correlation coefficients (ICC) between the given domains of the Hungarian version of the APFQ.

#### Internal consistency

Internal consistency of domains was analysed by McDonald’s omega (ω) coefficient. The ω-value gives a more accurate estimate of reliability than the classical Cronbach’s α-value; it is in fact a more general form of the α-value, which accounts for the factor loadings in confirmatory factor analysis. Values > 0.7 suggest acceptable internal consistency of the items [28].

#### Statistical analysis

Data were analysed with the SPSS 26 (IBM Corp.) and JASP statistical software. The appropriateness of the data to the normal distribution was examined with the Kolmogorov-Smirnov test. Chi-square test was used for categorical variables and Mann-Whitney U test for data with non-normal distribution. Spearman’s correlation analysis was used for evaluating the correlation between variables. Results are presented as numbers and medians with 25th and 75th percentiles or minimum and maximum values. The significance level was taken as *p* < 0.05.

Factorial validity of the APFQ was tested with confirmatory factor analysis (CFA, DWLS estimation). Goodness of fit was controlled on the basis of the following indices: χ2, comparative fit index (CFI), root mean square error of approximation (RMSEA) and standardized root mean square residual (SRMR). CFI ≥ 0.95, RMSEA ≤ 0.08 and SRMR ≥ 0.11 are acceptable results. In the case of the Bowel and Sex factors, which showed less than optimal fit, exploratory factor analysis (EFA, WLS estimation, ProMax rotation) was performed.

## Results

### Demographic data

A total of 294 participants were included in the validity and reliability study. The participants were divided into two main study groups: PFD (109 participants; POPQ stage 1. 29.4%, stage 2. 49.5%, stage 3. 13.8% and stage 4. 7.3%) and Control (CO; 185 participants). Members of PFD and CO groups significantly differed by age; the PFD participants were 8.73 years older (*p* < 0.001). The PFD and CO groups were not significantly different according to anthropometric and sociodemographic measures (BMI, place of residence and educational attainment). The number of the childbirths in the PFD group was significantly higher than in the control group (*p* < 0.001). The majority of participants had intellectual work and college or university diplomas and lived in urban areas. The main characteristics of the sample and data of the obstetric anamnesis are given in Table 1.

**Table 1:** Demographic data and obstetric medical history

	PFD group (n = 109)			CO group (n = 185)			
	Mean± SD	Min	Max	Mean± SD	Min	Max	*p* value
Age	54.91 ± 12.84	28	85	46.18 ± 11.64	20	82	< 0.001
BMI	25.90 ± 4.11	17.2	39.0	25.63 ± 5.38	16.7	52.6	0.247
Place of residence	n	%		n	%		*p* value
Capital	10	9.3		26	14.1		0.178
City	82	75.9		125	67.9		
Village	16	14.8		33	17.9		
Profession	n	%		n	%		*p* value
Physical work	38	35.5		54	29.5		0.001
Intellectual work	46	43.0		114	62.3		
Pensioner	23	21.5		15	8.2		
Educational attainment	n	%		n	%		*p* value
Vocational school	3	2.8		6	3.3		0.429
Secondary vocational school	30	27.5		44	23.9		
Secondary grammar school	17	15.6		19	10.3		
Higher education	59	54.1		115	62.5		
	PFD group			CO group			
	Median	Min	Max	Median	Min	Max	*p* value
Parturition	2.00	0	6	2.00	0	7	< 0.001
Vaginal childbirth	2.00	0	6	1.00	0	7	< 0.001
Caesarean section	0.00	0	1	0.00	0	3	0.019
Forceps delivery	0.00	0	1	0.00	0	1	0.267
Vacuum childbirth	0.00	0	1	0.00	0	2	0.924
Perineal injury/rupture	0.00	0	2	0.00	0	3	0.347
Episiotomy	1.00	0	4	0.00	0	4	< 0.001

Chi-square test, Mann-Whitney U test

### Reliability

In terms of the concerned psychometric properties, the internal consistency analysis showed a McDonald’s ω > 0.7 in all domains (Table 2).

**Table 2:** Reliability analysis between the Australian Pelvic Floor Questionnaire subscales

APFQ domains	McDonald’s ω (95% confidence interval)	McDonald’s ω if item dropped	Item-rest correlation
Bladder	0.888 (0.863-0.907)	0.865-0.891	0.211-0.757
Bowel	0.790 (0.735-0.831)	0.743-0.804	0.171-0.610
Prolapse	0.895 (0.871-0.916)	0.779-0.898	0.366-0.821
Sexual function	0.738 (0.673-0.783)	0.616-0.760	0.084-0.659

The test-retest reliability also showed strong and significant correlations between the given domains. Intraclass correlation coefficient between test and retest was in Bladder domain 0.83 (95% CI, 0.68-0.92), *p* < 0.001; in Bowel domain 0.92 (95% CI, 0.84-0.96), *p* < 0.001; in Prolapse domain 0.96 (95% CI, 0.92-0.98), *p* < 0.001; in Sexual function domain 0.87 (95% CI, 0.71-0.94), *p* < 0.001).

### Validity

#### Content/face validity

The rate of missing data after administration of all questionnaires was (Bladder 0.68-3.40%; Bowel 2.38 3.74%; Prolapse 3.06-3.74%; Sexual function in sexually active participants 2.34-4.21%) with respect to the two groups.

#### Discriminant validity

Clinically important and statistically significant differences of the bladder, bowel, sexual function and prolapse domain scores were noted between the two compared groups (Table 3).

**Table 3:** Australian Pelvic Floor Questionnaire results of control and Pelvic floor disorder groups

APFQ scores	CO group			PFD group			Z score	*p* value
	Median	25p*	75p*	Median	25p*	75p*		
APFQ Bladder	1.11	0.44	2.22	2.00	1.11	3.44	-5.294	< 0.001
APFQ Bowel	1.48	0.74	2.22	1.85	1.11	2.59	-2.051	0.040
APFQ Prolapse	0.00	0.00	0.00	3.33	2.00	5.33	-12.806	< 0.001
APFQ Sexual function	1.36	0.91	2.73	2.27	0.91	3.64	-3.182	0.001

Mann-Whitney test; APFQ Bowel: without questions 16 and 17; APFQ Sexual function: only sexually active participants, *25p = 25th percentile, 75p = 75th percentile

#### Convergent validity

In the total study population, strong correlations were observed between the APFQ bladder domain and the ICIQ-UI SF total score; the APFQ prolapse domain scores and POPQ status; the PISIQ-IR sexually active (global quality rating of sexual quality) and APFQ sexual function score (Table 4), which support good convergent validity of APFQ.

**Table 4:** Results of convergent validity

	Median	25p^***^	75p^***^	Spearman’s correlation coefficient	*p* value
ICIQ scores^*^(*n* = 109)	4.00	0.00	10.00	0.584	< 0.001
APFQ Bladder scores*(*n* = 109)	2.00	1.11	3.44		
POP Q stage*(*n* = 109)	2.00	1.00	2.00	0.339	< 0.001
APFQ prolapse scores*(*n* = 108)	3.33	2.00	5.33		
PISIQ-IR Sexually active: arousal, orgasm (*n* = 201)	75.00	63.00	81.00	0.518	< 0.001
APFQ Sexual function scores^**^(*n* = 205)	1.82	0.91	2.73		
PISIQ-IR Sexually active: condition-specific impacts on activity (*n* = 185)	100.00	83.00	100.00	0.567	< 0.001
APFQ Sexual function scores^**^(*n* = 205)	1.82	0.91	2.73		
PISIQ-IR Sexually active: partner-related impacts (*n* = 191)	89.00	78.00	100.00	0.269	< 0.001
APFQ Sexual function scores^**^(*n* = 205)	1.82	0.91	2.73		
PISIQ-IR Sexually active: sexual desire (*n* = 196)	58.00	50.00	67.00	0.103	0.157
APFQ Sexual function scores^**^(*n* = 205)	1.82	0.91	2.73		
PISIQ-IR Sexually active: condition-specific impact on sexual quality (*n* = 187)	92.00	75.00	100.00	0.553	< 0.001
APFQ Sexual function scores^**^(*n* = 205)	1.82	0.91	2.73		
PISIQ-IR Sexually active: global quality rating of sexual quality (*n* = 194)	73.00	53.00	100.00	0.375	< 0.001
APFQ Sexual function scores^**^(*n* = 205)	1.82	0.91	2.73		

*PFD group; **sexually active; ***25*p* = 25th percentile, 75*p* = 75th percentile

#### Factorial validity

Table 5 shows that the Bladder and Prolapse factors of APFQ fit well to the structure of our data, which proves the one-dimensionality of this scales. However, the Bowel and Sexual function scales fit less than optimally. These results are in line with McDonald’s ω values for the given scales.

**Table 5:** Fit indices of the Australian Pelvic Floor Questionnaire factors

APFQ	Χ^2^	df	p value	CFI	TLI	RMSEA (90% CI)	SRMR
Bladder	127.41	90	0.006	0.980	0.976	0.039 (0.022-0.054)	0.091
Bowel	56.89	26	< 0.001	0.936	0.912	0.065 (0.042- 0.088)	0.088
Prolapse	3.44	5	0.632	1.000	1.010	0.000 (0.000- 0.068)	0.110
Sexual function	16.93	5	0.005	0.918	0.836	0.109 (0.055- 0.169)	0.090

*CFI*, Comparative Fit Index; *TLI*, Tucker-Lewis Index; *RMSEA*, root mean square error of approximation; *CI*, confidence interval; *SRMR*, standardized root mean square residual

To clear the structure of the Bowel and Sexual function factors, exploratory factor analysis was performed, which provided the following results: The items of the Bowel scale are settled in a two-factor structure: the first factor consists the items (18-20, 24-26) of peristalsis symptoms, the second factor the items (21-23) of incontinence. The items of the Sexual function scale cannot outline a homogeneous construct. The items 35, 38 and 41 have < 0.4 loadings.

During the linguistic validation, the authors strived for not only simple translation of the original questionnaires, but also development of conceptually equivalent and culturally appropriate versions adapted. Internal consistency was analysed, applying McDonald’s ω that was > 0.7 for every dimension of the APFQ-H. (In the original article, the internal consistency was similar: bladder function 0.72; bowel function 0.82; sexual function 0.81; POP 0.95). APFQ-H is an instrument with strong internal consistency showing moderate to good correlation among items.

## Discussion

APFQ has been translated and validated in seven countries, and it is also used to clarify PFD in patients with different medical conditions, such as pregnant and postpartum women, patients with colorectal cancer and pre- and postoperative conditions [13–17, 20, 29, 30]. Currently there are no available data on Hungarian women’s PFD and quality of life, so it would be very important to have a validated, self-administered questionnaire, which evaluates bladder, bowel, prolapse and sexual function domains, instead of separate questionnaires, reducing participant burden in both research and clinical settings and giving more appropriate information about patient condition during shorter examination times. (The self-administered version is an appropriate instrument of choice in outcome research as well.) Therefore, the aim of the study was to translate, validate and adapt the self-administered APFQ questionnaire into Hungarian to assess its reliability and validity among healthy women and patients with POP. In the present study, the response rate of > 99% for the questions confirms content validity; only the sexual function domain showed a lower rate, which was satisfactory (95.79%) as well despite the taboo nature of the subject. Intraclass correlation coefficients for the domain scores were in the range 'acceptable to excellent', i.e., > 0.7, except sexual function. Confirmatory factor analysis also showed lower fit scores in the sexual function domain. The results of the exploratory factor analysis suggest that the items of this scale assess different aspects of sexual dysfunction (disturbance of lubrication, vaginal distention, coital pain, coital incontinence) that are relatively independent of each other and therefore do not form a homogeneous scale. Astepe et al. found similar results in their study in the Turkish female population [14].

The results of the APFQ were weakly to moderately correlated with those of other symptom questionnaires and examination results (ICIQ-UI SF, PISQ-IR, POPQ). Our results provide convincing evidence for the convergent validity of APFQ-H. Other validation studies established that PFD scales correlated moderately to strongly with the POP and Bladder dimensions of the APFQ [14, 16].

Discriminant validity presented a significant difference for all dimensions in this study similarly to other APFQ validations. In a previous study, a different result was found in the sexual domain [14].

This study has several limitations. First, most of the respondents reside in urban areas and more than half of the participants had high educational background. The population we studied was neither representative nor randomized. Another limitation is that there was no validated questionnaire in the Hungarian language covering anal incontinence. Furthermore, there was no significant result for the desire in sexual function domain in this study. The results of sexual function of patients and control group were also not significant in a previous similar validation study [14]. Nevertheless, further evaluation may be required to determine the prevalence and impact of sexual dysfunction with other tools in women with POP in Hungary, particularly in terms of desire.

Despite these limitations, we believe that the APFQ-H is a valid and reliable instrument that should be widely used in both clinical and research settings to measure PFDs in Hungarian women. To provide effective support for women with PFDs, with particular attention to sexual desire, we need to conduct more research that explores the multidimensional aspects of PFDs and correlative factors.

The Hungarian version of the self-administered APFQ assesses comprehensively the domains of bladder, bowel and sexual function and prolapse symptoms, which had satisfactory reliability, validity and responsiveness.

## Conclusion

This was the first study which translated the self-administered, four-domain, complex questionnaire from English to Hungarian and validated it using a Hungarian-speaking female study sample. At present, in Hungary other questionnaires assess only some aspects of PFD. The Hungarian version of the self-administered APFQ is a reliable and valid instrument for evaluating symptom severity and impact of PFDs on the QOL. It will enable researchers and clinicians from Hungary to use a well-validated instrument to assess PFDs. (APFQ-H can be found in the Appendix.)

## Supplementary information

ESM 1 (DOCX 24 kb)
