# Supplementary material for: Validation and translation of the Hungarian version of the Australian Pelvic Floor Questionnaire (APFQ-H)
Source: Int Urogynecol J. 2022 Aug 16;34(6):1187–94. doi: 10.1007/s00192-022-05322-2 (PMC10238311; doi:10.1007/s00192-022-05322-2)
Supplement: Supplementary file 1 — (DOCX 24 kb) [file 192_2022_5322_MOESM1_ESM.docx]

**Appendix**

**Hungarian Version of Australian Pelvic Floor Questionnaire, APFQ-H**

**Kérem, X-szel jelölje be az Önre legjellemzőbb választ az elmúlt egy hónap tünetei alapján.**

Név vagy kód: _____________________________ Születési idő: ______________________________

**Hólyag funkciók**

1) **Egy nap** hányszor üríti vizeletét?

0 7 vagy kevesebb alkalommal

1 8-10 alkalommal

2 11-15 alkalommal

3 több mint 15 alkalommal

2) **Éjszaka** hány alkalommal szükséges felkelnie vizeletét üríteni?

0 0 - 1

1 2 alkalommal

2 3 alkalommal

3 több mint 3 alkalommal

3) Előfordult már Önnél akaratlan vizeletvesztés **éjszaka**?

0 soha

1 időnként – kevesebb mint heti 1 alkalommal

2 gyakran – heti 1 vagy több alkalommal

3 mindig – minden éjjel

4) Előfordult már Önnel, hogy **azonnal** rohannia / **sietnie** kellett a mellékhelyiségbe **vizelési inger jelentkezésekor**?

0 soha – tudom tartani

1 időnként – kevesebb mint heti 1 alkalommal

2 gyakran – heti egynél több alkalommal

3 minden nap

5) Előfordul, hogy elcseppen a vizelete, amikor a mellékhelyiségbe siet?

0 soha

1 időnként – kevesebb mint heti 1 alkalommal

2 gyakran – heti egynél több alkalommal

3 minden nap

6) Előfordul Önnel, hogy **elcseppen** a vizelete **köhögés, tüsszentés, nevetés vagy fizikai aktivitás** során?

0 soha

1 időnként – kevesebb mint heti 1 alkalommal

2 gyakran – heti egynél több alkalommal

3 minden nap

7) Előfordul Önnel, hogy a **vizeletsugara gyenge, lassú** vagy a vizelet ürítése túl hosszú ideig tart?

0 soha

1 időnként – kevesebb mint heti 1 alkalommal

2 gyakran – heti egynél több alkalommal

3 minden nap

8) Előfordul Önnél, hogy úgy érzi **nem tudja teljes mértékben üríteni** húgyhólyagját?

0 soha

1 időnként – kevesebb mint heti 1 alkalommal

2 gyakran – heti egynél több alkalommal

3 minden nap

9) Előfordul Önnel, hogy **erőlködnie kell**, hogy húgyhólyagját üríteni tudja?

0 soha

1 időnként – kevesebb mint heti 1 alkalommal

2 gyakran – heti egynél több alkalommal

3 minden nap

10) Használ Ön **betétet vagy tisztasági betétet vizelet elcseppenés miatt**?

0 soha

1 csak elővigyázatosságból

2 csak fizikai aktivitás során vagy megfázás esetén

3 minden nap

11) Előfordul Önnel, hogy **kevesebb folyadékot fogyaszt**, csak hogy **elkerülje a vizelet elcseppenését**?

0 soha

1 csak ha valahova menni kell, vagy társasági esemény előtt

2 időnként

3 minden nap

12) **Gyakran** van Önnek **húgyúti fertőzése**?

0 nem

1 évente 1-3 alkalommal

2 évente 4-12 alkalommal

3 havonta több alkalommal

13) Előfordul, hogy **vizeletürítés közben fájdalmat érez** a **húgycsövében**, vagy **húgyhólyagjában**?

0 soha

1 időnként – kevesebb mint heti 1 alkalommal

2 gyakran – heti egynél több alkalommal

3 naponta

14) **Befolyásolja** Önt a **vizelet elcseppenés** a napi **rutin tevékenységekben** (pl.: szabadidő, társasági élet, alvás, bevásárlás stb.)?

0 egyáltalán nem

1 kissé

2 közepesen

3 nagyon

15) Mennyire **zavarja** Önt a hólyag problémája?

0 egyáltalán nem

1 kissé

2 közepesen

3 nagyon

Egyéb tünetek (pl.: ülés/járás problémák, egyéb fájdalom, hüvelyi vérzés stb.)?

.........................................................................................................................................................................................................

Kérdések 1-15 eredménye /45 =

**Bél/székletürítési funkciók**

16) Milyen **gyakran üríti székletét** általában?

0 naponta vagy két naponta

1 három naponta vagy ritkábban

2 heti egyszer vagy ritkábban

0 naponta többször

17) Milyen **állagú** a széklete általában?

0 puha 0 szilárd / jól formált

1 kemény / golyók

2 vizes / folyós

1 változó

18) Előfordul, hogy sokat kell **erőlködnie** ahhoz, hogy székletét üríteni tudja?

0 soha

1 időnként – kevesebb mint heti 1 alkalommal

2 gyakran – heti egynél több alkalommal

3 Naponta

19) Előfordul, hogy **hashajtót** kell használnia, ahhoz, hogy székletét üríteni tudja?

0 soha

1 időnként – kevesebb mint heti 1 alkalommal

2 gyakran – heti egynél több alkalommal

3 naponta

20) Előfordul, hogy **székrekedése** van?

0 soha

1 időnként – kevesebb mint heti 1 alkalommal

2 gyakran – heti egynél több alkalommal

3 naponta

21) Előfordult már Önnel, hogy nem tudta kontrollálni/**visszatartani szellentését**?

0 soha

1 időnként – kevesebb mint heti 1 alkalommal

2 gyakran – heti egynél több alkalommal

3 naponta

22) Előfordul Önnel, hogy elsöprő/**kibírhatatlan sürgető ingert** érez a **székletürítésre**?

0 soha

1 időnként – kevesebb mint heti 1 alkalommal

2 gyakran – heti egynél több alkalommal

3 naponta

23) Előfordul Önnel, hogy **akaratán kívül üríti székletét**, ha az **folyós állagú**?

0 soha

1 időnként – kevesebb mint heti 1 alkalommal

2 gyakran – heti egynél több alkalommal

3 naponta

24) Előfordul Önnel, hogy **akaratán kívül üríti székletét**, ha az **normál állagú**?

0 soha

1 időnként – kevesebb mint heti 1 alkalommal

2 gyakran – heti egynél több alkalommal

3 naponta

25) Előfordul, hogy úgy érzi, **nem tudja teljes mértékben üríteni székletét**?

0 soha

1 időnként – kevesebb mint heti 1 alkalommal

2 gyakran – heti egynél több alkalommal

3 naponta

26) Előfordul, hogy **ujjával nyomást kell gyakorolnia végbelére**, ahhoz, hogy **székletét üríteni tudja**?

0 soha

1 időnként – kevesebb mint heti 1 alkalommal

2 gyakran – heti egynél több alkalommal

3 naponta

27) Mennyire **zavarják** Önt **székletürítéssel kapcsolatos** problémái?

0 egyáltalán nem

1 kissé

2 közepesen

3 nagyon

Egyéb tünetek (pl.: fájdalom, nyákos folyás, végbél süllyedés stb.)?

.............................................................................................................................................................................

Kérdések 16-27 eredménye /34 =

**Hüvelyfali süllyedéses tünetek**

28) Érez **előesést/kiboltosulást** vagy **dudort** a hüvelyében?

0 soha

1 időnként – kevesebb mint heti 1 alkalommal

2 gyakran – heti egynél több alkalommal

3 naponta

29) Tapasztal **nyomó, húzó vagy nehézség érzést** a hüvelyében?

0 soha

1 időnként – kevesebb mint heti 1 alkalommal

2 gyakran – heti egynél több alkalommal

3 naponta

30) Előfordul Önnel, hogy **vissza kell nyomnia ujjával a hüvelyfali előboltosulást**, ahhoz, hogy **vizeletét** üríteni tudja?

0 soha

1 időnként – kevesebb mint heti 1 alkalommal

2 gyakran – heti egynél több alkalommal

3 naponta

31) Előfordul Önnel, hogy **vissza kell nyomnia ujjával a hüvelyfali előboltosulást**, ahhoz, hogy **székletét** üríteni tudja?

0 soha

1 időnként – kevesebb mint heti 1 alkalommal

2 gyakran – heti egynél több alkalommal

3 naponta

32) Mennyire **zavarja** Önt a hüvelyfali süllyedése/előesése?

0 egyáltalán nem 0 nincs süllyedésem

1 kissé

2 közepesen

3 nagyon

Egyéb tünetek (pl.: ülés/járás problémák, egyéb fájdalom, hüvelyi vérzés stb.)?

......................................................................................................................................................................................

Kérdések 28-32 eredménye /15 =

**Szexuális funkciók**

33) Aktív Ön szexuálisan? (Nem pontozzuk válaszát)

nem

kevesebb mint heti 1 alkalommal

heti egynél több alkalommal

naponta / a legtöbb napon

**Amennyiben Ön szexuálisan nem aktív, kérem, csak a 34-es és 42-es kérdésekre válaszoljon.**

34) Amennyiben Ön szexuálisan nem aktív, kérem mondja el miért. (Nem pontozzuk válaszát)

nincs partnerem

a partnerem nem képes szexuális aktivitásra

18 hüvelyi szárazság miatt

18 túl fájdalmas

18 hüvelyfali süllyedés / inkontinencia miatti szégyen

egyéb okok miatt

35) Megfelelő Önnél a hüvely természetes síkossága / nedvessége szexuális aktus alatt?

0 igen

1 nem

36) Szexuális aktus során a hüvelyben tapasztalt érzés:

0 normál / kellemes

1 minimális érzés

1 fájdalmas

3 nincs semmilyen érzés

37) Előfordul, hogy úgy érzi, hogy a hüvelye túl tág, vagy laza?

0 soha

1 néha

2 gyakran

3 mindig

38) Előfordul, hogy úgy érzi, hogy a hüvelye túl szűk?

0 soha

1 néha

2 gyakran

3 mindig

39) Előfordul Önnel, hogy fájdalmat tapasztal szexuális aktus során?

0 soha

1 néha

2 gyakran

3 mindig

40) Hol tapasztal fájdalmat szexuális aktus során?

0 nincs fájdalmam

1 a hüvely bemeneténél

1 mélyen / a medencében

2 a hüvely bemeneténél és a medencében is

41) Előfordul Önnél akaratlan vizeletvesztés szexuális aktus során?

0 soha

1 néha

2 gyakran

3 mindig

42) Mennyire zavarják Önt ezek a szexuális problémák?

0 egyáltalán nem 0 nincs problémám

1 kissé

2 közepesen

3 nagyon

Egyéb tünetek (pl.: közösüléskor szellentés vagy széklet inkontinencia, vaginizmus, stb.)?

......................................................................................................................................................................................................

Kérdések 33-41 eredménye /21 =

Kitöltés dátuma: ________________________________
